# Increase in conduction velocity in myelinated nerves due to stretch – An experimental verification

**DOI:** 10.3389/fnins.2023.1084004

**Published:** 2023-04-17

**Authors:** Sabrina Sharmin, Mohammad Abu Sayem Karal, Zaid Bin Mahbub, Khondkar Siddique-e Rabbani

**Affiliations:** ^1^Department of Physics, Bangladesh University of Engineering and Technology, Dhaka, Bangladesh; ^2^Department of Arts and Sciences, Ahsanullah University of Science and Technology, Dhaka, Bangladesh; ^3^Department of Mathematics and Physics, North South University, Dhaka, Bangladesh; ^4^Department of Biomedical Physics and Technology, University of Dhaka, Dhaka, Bangladesh

**Keywords:** nerve conduction velocity, ulnar nerve, elbow flexion, nerve stretch, myelinated nerve, nodes of Ranvier

## Abstract

**Background:**

Based on published experimental evidence, a recent publication revealed an anomalous phenomenon in nerve conduction: for myelinated nerves the nerve conduction velocity (NCV) increases with stretch, which should have been the opposite according to existing concepts and theories since the diameter decreases on stretching. To resolve the anomaly, a new conduction mechanism for myelinated nerves was proposed based on physiological changes in the nodal region, introducing a new electrical resistance at the node. The earlier experimental measurements of NCV were performed on the ulnar nerve at different angles of flexion, focusing at the elbow region, but left some uncertainty for not reporting the lengths of nerve segments involved so that the magnitudes of stretch could not be estimated.

**Aims:**

The aim of the present study was to relate NCV of myelinated nerves with different magnitudes of stretch through careful measurements.

**Method:**

Essentially, we duplicated the earlier published NCV measurements on ulnar nerves at different angles of flexion but recording appropriate distances between nerve stimulation points on the skin carefully and assuming that the lengths of the underlying nerve segment undergoes the same percentages of changes as that on the skin outside.

**Results:**

We found that the percentage of nerve stretch across the elbow is directly proportional to the angle of flexion and that the percentage increase in NCV is directly proportional to the percentage increase in nerve stretch. Page’s L Trend test also supported the above trends of changes through obtained *p* values.

**Discussion:**

Our experimental findings on myelinated nerves agree with those of some recent publications which measured changes in CV of single fibres, both myelinated and unmyelinated, on stretch. Analyzing all the observed results, we may infer that the new conduction mechanism based on the nodal resistance and proposed by the recent publication mentioned above is the most plausible one to explain the increase in CV with nerve stretch. Furthermore, interpreting the experimental results in the light of the new mechanism, we may suggest that the ulnar nerve at the forearm is always under a mild stretch, with slightly increased NCV of the myelinated nerves.

## Introduction

Using a recently developed nerve conduction parameter called ‘Distribution of F-Latency (DFL)’, which approximates a mirror image of the relative ‘Distribution of Conduction Velocity (DCV)’ of A-alpha fibres of peripheral nerves (Rabbani et al., 2007; Rabbani, 2011), a head bending experiment suggested immediate changes in conduction velocity (CV) of motor fibres of the median nerve at the neck which again reverted back immediately on head straightening (Rahman, 2008; Mahbub, 2014; Sharmin and Rabbani, 2016). Such quick changes in CV cannot be explained based on existing concepts of nerve fibre conduction. Following this cue, Rabbani (2018) found supporting evidence in earlier works (Harding and Halar, 1983; Sattari and Emad, 2007) who reported instantaneous and reversible increases in NCV from full extension to full flexion of ulnar nerve at the elbow. However, while trying to explain these findings, Rabbani (2018) came across an anomaly and proposed a new nerve conduction model in order to resolve this. This is presented below giving a brief outline of the existing accepted concepts on nerve conduction first.

**Figure 1:**
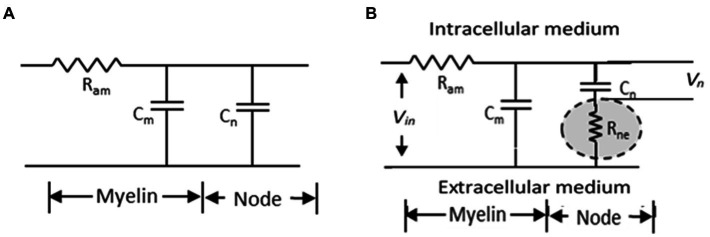
Simplified electrical model of a myelinated nerve fibre. **(A)** traditional model **(B)** proposed model (Rabbani, 2018).

Nerve conduction velocity (NCV) for a nerve trunk or conduction velocity (CV) of a nerve fibre have been measured over the past century in different types of living organisms, in vertebrates and non-vertebrates, in both myelinated and non-myelinated nerves (Hoffmeister et al., 1991; Hennessey et al., 1994; Qiao et al., 2012; Preston and Shapiro, 2015; Walsh et al., 2015). From these studies, it was established that CV is higher for nerve fibres with larger diameters for the same type of fibres (i.e., either non-myelinated or myelinated) (Waxman, 1980; Hobbie and Roth, 2007). Electrical parametric models have also been developed over the past century to support these findings (Hodgkin and Huxley, 1952; McIntyre et al., 2002; Kolaric et al., 2013; Brown and Hamann, 2014) based on which a cable theory was developed for myelinated fibres and a simplified model is shown in Figure 1A. According to this model, the time constant (*τ*) defining the delay for propagation of action potentials is given by the product of the axonal resistance between two successive nodes (*R*_am_) and the sum of the capacitances of the nodal region (*C*_n_) and the internodal (*C*_m_) regions, as:


(1) $$\tau =R_{\mathrm{am}}\left(C_{m}+C_{n}\right)$$

This model ignores any activity of leaky channels, particularly during the progression from the resting potential to the threshold potential for generation of an action potential. Since CV is supposed to be inversely proportional to *τ* a detailed analysis of the parameters of Equation 1 supported the previous concept that CV is proportional to the diameter of a nerve fibre.

The anomaly that Rabbani (2018) observed came from published experimental works (Harding and Halar, 1983; Sattari and Emad, 2007) who obtained instantaneous and reversible increases of about 20% in NCV in an approximately 10 cm length of the ulnar nerve around the elbow due to elbow flexing from 0° (straight elbow) to about 135° (full flexion). There was a systematic linear increase over this range at intermediate angles of flexion. Although such a systematic increase of NCV with angle of flexion was observed for a large number of individuals in both these works, these authors assumed that the change occurred due to possible sliding of the ulnar nerve at the elbow leading to measurement errors. They also did not publish actual lengths used for the measurements at different angles of flexion. Their main purpose was to find out the optimum flexing angle so that the NCV measured across the elbow segment is the same for the NCV in the below-elbow segment. It was argued by Rabbani (2018) that since the ulnar nerve winds around the medial epicondyle at the elbow, flexion at elbow will stretch the ulnar nerve, which will also decrease the fibre diameters. He found support to this latter claim in Thoirs et al. (2008), which reported a reduction of about 20% in the ulnar nerve diameter at the elbow from full extension to full flexion using ultrasound scanning measurements. Now, previously mentioned studies (those that led to Equation 1) suggested that the CV should decrease for a reduction of diameter. On the other hand, the above mentioned experiments by Harding and Halar (1983) and Sattari and Emad (2007), in light of the findings by Thoirs et al. (2008), give a completely opposite finding. These essentially imply that CV increased while the axonal diameter decreased through the maneuvre, which is therefore, an anomaly. Since conventionally measured NCV on ulnar nerves using surface electrodes involve only large diameter myelinated nerve fibres of the A-alpha group (Kimura, 1989), it was suggested by Rabbani (2018) that this anomaly, not noticed before, occurs in myelinated nerve fibres only.

To explain the increase in NCV of a myelinated nerve on stretching, Rabbani (2018) invoked published electron micrographic images and models of the nodal region in myelinated nerves which suggested that protrusions from myelin sheath layers from the two sides of a node formed an interdigitated interface with a very narrow gap in between. He suggested that this gap should be very tight because of the elastic pull of the endoneurium that covers each myelinated nerve fibre and will pose a very large resistance to movement of ionic charges between the immediate outer surface of the axonal membrane at the node and the extra cellular fluid outside the myelin cover. In fact these ionic movements constitute the node to node current which is needed for nerve signal propagation. He named this resistance as ‘node to extracellular resistance (*R*_ne_)’ and put forward a modified cable network for neural conduction as shown in Figure 1B showing the positioning of the newly introduced resistance with respect to the previous cable network. It is to be noted that it is the potential *V*_n_ across the nodal capacitance *C*_n_ which determines initiation of an action potential. Rabbani (2018) argued that the axon has a diameter of around 10 *μ*m while the gaps of the interdigitated fingerlike processes at the node will be much smaller, expected to be in the range of tens of nm. Besides, the ions have to move across a zigzag path, through many bends and obstacles increasing the effective length. Therefore, *R*_ne_ should be much greater than *R*_am_ and the delay for propagation would be dominated by the new time constant *τ*_n_ involving both *R*_ne_ and the nodal capacitance *C*_n_ as,


(2) $$\tau_{n}=R_{\mathrm{ne}}C_{n}\;$$

As the nerve is stretched, the interdigitated protrusions of the myelin sheaths from two sides of a node will be pulled apart increasing the gap width and so *R*_ne_ would decrease sharply, thereby increasing the CV overriding other factors that may oppose this change. Again as soon as the stretching force is withdrawn, the gap width will decrease and CV will come back to its relaxed value, and this will happen immediately, which was also observed in the experiments referred to earlier. This then explains the observed anomaly, argued Rabbani (2018). However, this work did not provide any direct quantitative experimental verification of the observed anomaly except drawing from the results of previous published works.

The motivation for the present work arose from this lack of quantitative experimental evidence mentioned above. The experiments were carried out in a way similar to those of Harding and Halar (1983) and Sattari and Emad (2007) mentioned earlier but the focus was on the measurement and recording of the distances of the stimulating points on the skin at different angles of flexion, in order to relate these values to possible nerve stretch and in turn to changes in NCV. However, the authors recognize that there may be other factors affecting NCV on stretching of the ulnar nerve which will be discussed later.

### Experimental design and methods

For simplicity, this work makes the following assumptions:

1. The nerve segment around the medial epicondyle undergoes only stretch from full extension to full flexion, there is no slack to be removed. Therefore, any change in measured NCV within this segment is due to nerve stretch only. Of course, one should keep in mind that there may be removal of slacks in the nerve trunk in the upper arm region (Schuind et al., 1995; Novak et al., 2012) which may introduce errors at low angles of flexion. This will be discussed later.
2. The nerve lies at the same depth with respect to the skin in the measurement region so that the percentage of nerve stretch will be the same as the percentage of stretch of the corresponding skin segment.

It is understood that the length of the skin segment will be somewhat greater than the length of the nerve segment underneath, but the above 2^nd^ assumption will be valid if no slack in the nerve is to be removed. Therefore, it is the percentage change in NCV with respect to the percentage change in nerve length which will be of relevance and importance, not the absolute values. However, the absolute values of NCV were used to have a preliminary visualization of the changes and their statistical significance, assuming that the nerve segment lengths and the corresponding distances on the skin are the same.

**Figure 2:**
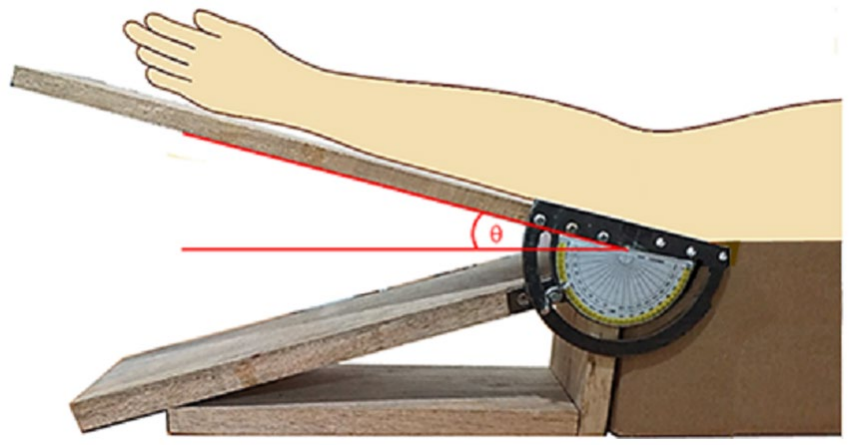
Homemade gadget to measure NCV due to stretching at different angles of elbow flexion.

In order to carry out the experimental investigation, a homemade gadget was designed and made as shown in Figure 2. The elbow of a subject could be fixed at specific angular positions, with appropriate angular markings as indicated by the schematics superimposed on the photograph.

A total of 44 nerves from the left and right hands of 22 healthy subjects (17 male, 5 female) of mean age 30 years, without any diagnosed neurological disorder, were examined. The subjects volunteered to participate in the study according to their convenience. Their written consent for the study, according to the protocol of the informed consent of the Bangladesh Medical Research Council (BMRC), were obtained.

For measurement of NCV, a Nicolet EDX system (Natus Neurology, Middleton, WI, USA) was used. All the measurements were carried out at room temperature (~25°C). The stimulus current was increased gradually from 10 mA upwards to ensure supramaximal stimulation. The duration of each stimulus was set at 0.1 ms. The other measuring set up were: amplifier range 100 mV, sampling rate 48 kHz, sweep duration 20 ms, low cut-off frequency 0.6 Hz and high cut-off frequency 10 kHz.

**Figure 3:**
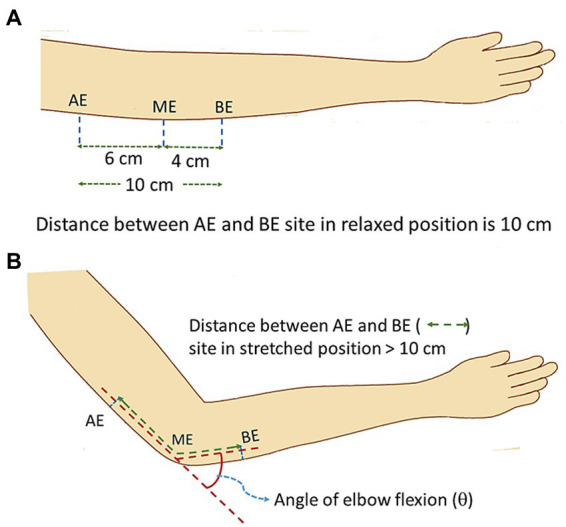
Schematic diagram of elbow position during experiment. **(A)** relaxed position, **(B)** flexed position (stretched ulnar nerve).

Figure 3A shows the schematic diagram of elbow position at a flexing angle of 0° (relaxed state) while Figure 3B shows the same at a flexed position, at an angle *θ*. The positions AE (above elbow), ME (mid elbow – located in reference to the Medial Epicondyle and the Olecranon Process, OP) and BE (below elbow) indicate the positions around the elbow used as references to study the changes in the NCV on elbow flexion. In order to relate the measurements to those of Harding and Halar (1983) and Sattari and Emad (2007) discussed earlier, the same segment lengths were used. The AE-BE segment at relaxed state (Figure 3A) was chosen to be 10 cm, the point AE at 6 cm proximal to ME and the point BE at 4 cm distal to ME. These positions were marked on the skin using a pen. With the change of angle of flexion, the distances of the AE-ME and BE-ME segments (from the respective pen markings to the OP) increased which were carefully measured. Adding both these segment lengths for each angle of flexion the total length of the AE-BE segment was obtained.

**Figure 4:**
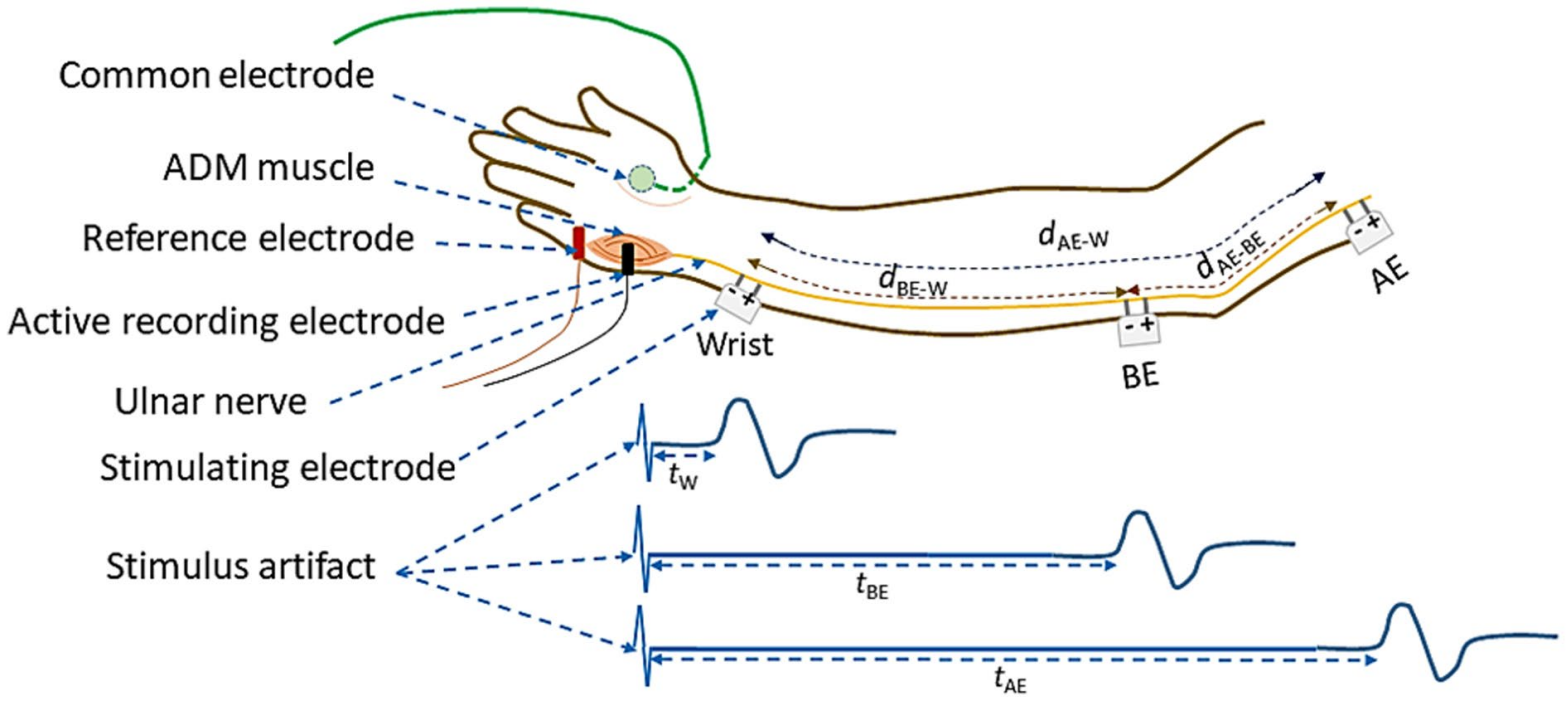
Schematic diagram of electrode arrangement for the measurement of NCV of the ulnar nerve between different nerve segments.

The schematic diagram of electrode arrangement for measurement of NCV is presented in Figure 4. The active recording electrode was placed on the prominent part of the abductor digiti minimi (ADM) muscle. The reference electrode was placed over the hypothenar tendon at the metacarpophalangeal (MCP) joint of the fifth digit. The inter-electrode distance was kept constant at 4 cm from center to center. The common or neutral electrode was placed on the dorsum of the hand. Electrical stimulation was applied at three positions: i) wrist (W), about 7 cm proximal from the active recording electrode, ii) below elbow (BE), and iii) above elbow (AE). Each of these produced corresponding compound muscle action potentials as shown schematically in Figure 4. As mentioned before, the distance between BE and AE was chosen to be 10 cm, 4 cm distal and 6 cm proximal to ME, at 0° angular position of elbow (Figure 3A), which was taken to be the relaxed condition. Motor NCVs were determined for three segments along the course of ulnar nerve and categorized as the velocities *NCV*_BE-W_, *NCV*_AE-W_ and *NCV*_AE-BE_, where the subscripts indicate the respective segments. The measurements were taken from subjects sitting on a chair in a relaxed position with the hand placed on the wooden frame to fix the elbow at desired angular position shown in Figure 2. For each angle of flexion, the changed distances of the AE-ME and ME-BE segments (from positions of the pen markings, Figure 3B) were measured using a measuring tape to give the length *d*_AE-BE_ of the AE-BE segment (Figure 4) since the change in length of this segment is the important one for this experiment. For measurement of *NCV*_BE-W_, the appropriate distance *d*_BE-W_ was measured while for *NCV*_AE-W_, the corresponding distance *d*_AE-W_ was obtained summing the two measured distances *d*_AE-BE_ and *d*_BE-W_ (Figure 4).

The latency value *t*_W_ corresponds to the time between the stimulus applied to the wrist and the onset of the CMAP (combined motor action potential) as recorded from the ADM muscle of the palm and as shown in Figure 4. Similarly, *t*_BE_ and *t*_AE_ are the corresponding latencies obtained due to the stimulus applied at BE and AE, respectively. The latency *t*_W_ includes the uncertain latency at the neuro-muscular junction which was subtracted from *t*_BE_ and *t*_AE_ to obtain the corresponding NCV of the nerve segments BE-W and AE-W, respectively. The NCV for the AE-BE segment was obtained through a subtraction of the above latencies. The latency values were obtained directly from the acquired data in the computer. The respective NCV values were obtained from the above latency values and the corresponding measured distances along nerve between the two respective stimulating electrodes, according to the formulae given below:


(3) $$NCV_{AE-W}=\frac{d_{AE-W}}{t_{AE-W}}$$

(4) $$NCV_{BE-W}=\frac{d_{BE-W}}{t_{BE-W}}$$

(5) $$NCV_{AE-BE}=\frac{d_{AE-BE}}{t_{AE-BE}}$$

NCVs as above were determined for ulnar nerves of both hands at five specific angles of elbow flexion, 0, 45, 90, 110 and 135 degrees, respectively.

### Statistical analysis

Firstly, the experimental results were analyzed to determine whether the NCVs indeed increased in a systematic way with angle of flexion, or if these varied randomly. For this Page’s L Trend test (Page, 1963) was performed to test the statistical significance of each of the experimental parameters, *NCV*_BE-W,_ *NCV*_AE-W_ and *NCV*_AE-BE_ respectively.According to this test the null hypothesis was set as (subscripts indicating the angles of flexion),

*Null Hypothesis, Ho: NCV*_0_ = *NCV*_45_ = *NCV*_90_ = *NCV*_110_ = *NCV*_135_, against the systematic incremental hypothesis,

*Systematic Incremental hypothesis, Hi: NCV*_0_ < *NCV*_45_ < *NCV*_90_ < *NC*V_110_ < *NCV*_135_.

As will be shown in the next section that the null hypothesis holds for the BE-W segment while it is rejected for the AE-W and AE-BE segments, which means the systematic incremental hypothesis holds for these latter two. However, the main focus of the present work is the AE-BE segment of the nerve around the elbow. As mentioned before, the percentage of nerve stretch for this segment is assumed to be the same as the percentage of stretch of the corresponding outside skin segment. Furthermore, as presented in the next section, the relationship between the measured percentage of stretch of the segmental distance *d*_AE-BE_ and the angle of flexion was found to be linear. Therefore, for subsequent analyses the percentage changes in *NCV*_AE-BE_ were studied in terms of the percentage of stretch of *d*_AE-BE_ directly, instead of the angle of flexion. All results are presented in the next section.

## Results

**Figure 5:**
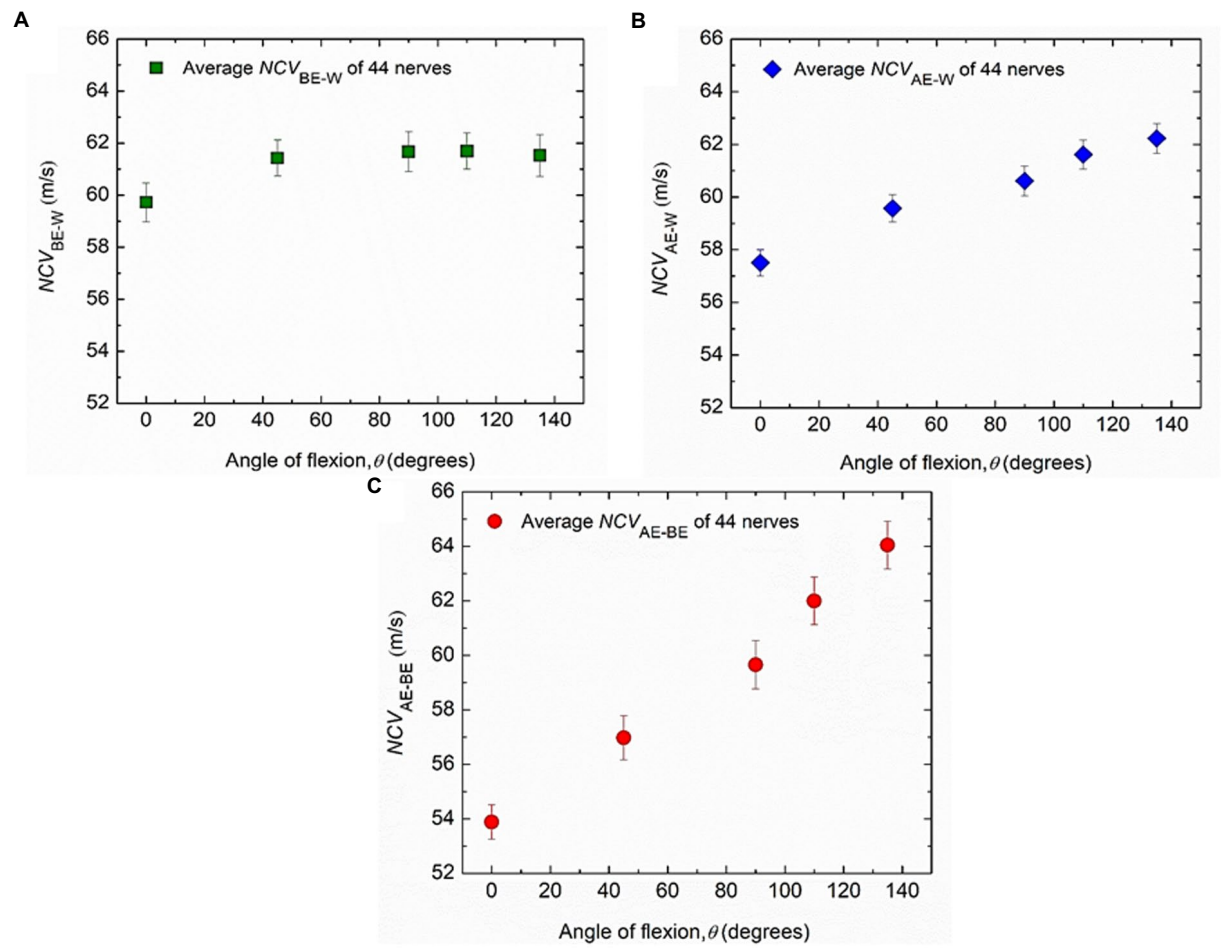
Experimental changes of average values of conduction velocities. **(A)** *NCV*_BE-W_, **(B)** *NCV*_AE-W_ and **(C)** *NCV*_AE-BE_ corresponding to different angles of elbow flexion from 44 nerves of both hands of 22 subjects. Average values and standard errors of means are shown for each experimental point.

Firstly, the experimental results for the nerve conduction velocities *NCV*_BE-W_, *NCV*_AE-W_ and *NCV*_AE-BE_, averaged over those for all the 44 nerves from both hands of 22 subjects are presented against the angle of elbow flexion in Figure 5. The standard errors of means are also indicated. It may be observed that *NCV*_BE-W_ remains essentially unchanged for different angles of flexion, at a value slightly more than 61.5 m/s (Figure 5A) although there is a small reduced value, slightly less than 60 m/s for a straight hand (0°). Figure 5B indicates a steady but small increase in *NCV*_AE-W_, from about 57.5 m/s to about 62.5 m/s for angles of flexion from 0° to 135°. Finally, Figure 5C shows that *NCV*_AE-BE_ increases almost linearly from about 54 m/s to about 64 m/s for angles of flexion from 0° to 135°. It is interesting to note that for angles of flexion less than about 110°, *NCV*_AE-BE_ is lower than the near constant value of 61.5 m/s for *NCV*_BE-W_. An attempt to explain this phenomenon will be made in the light of the new mechanisms (Rabbani, 2018) in the discussion section.

**Figure 6:**
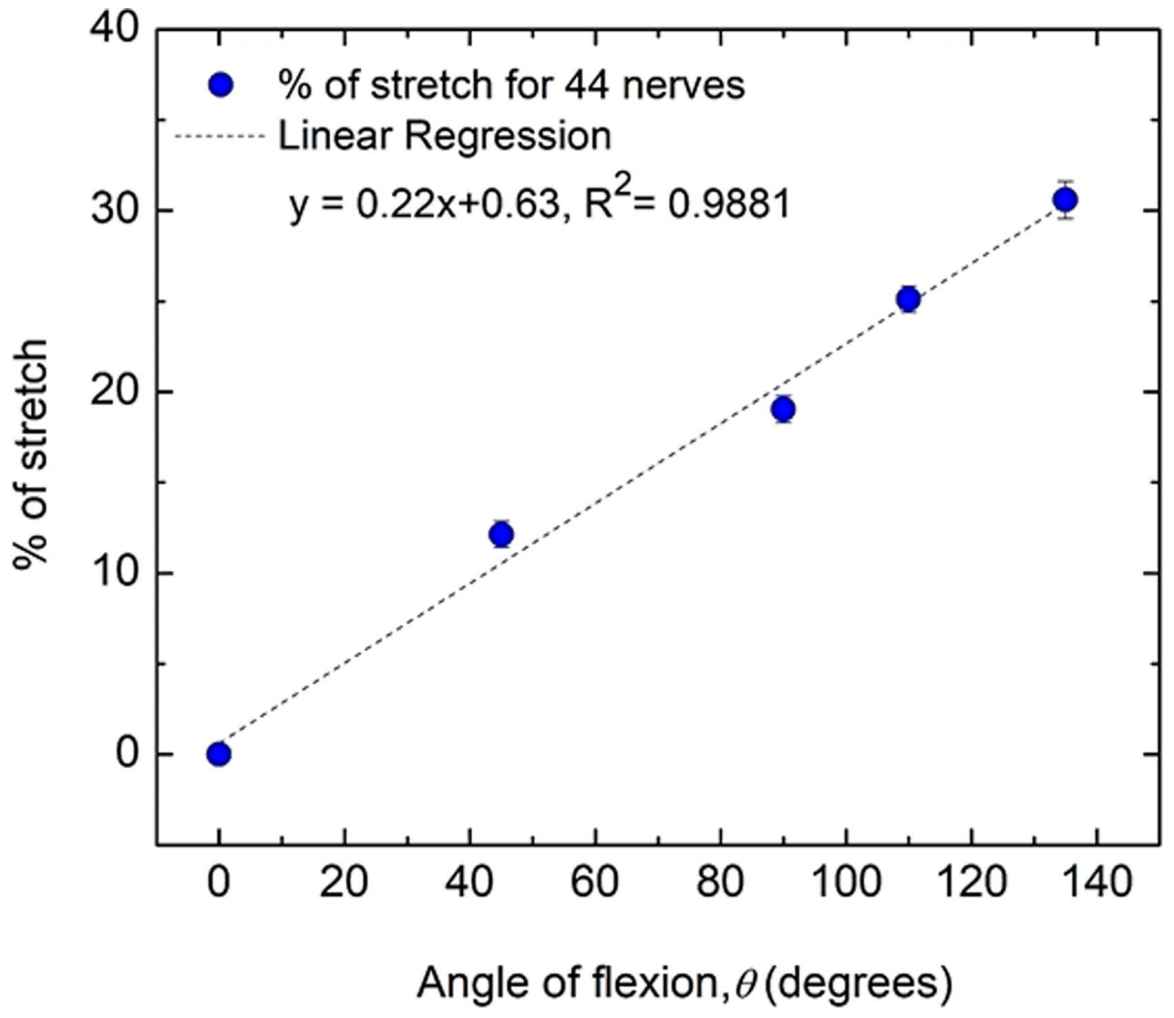
Percentage of nerve stretch with flexion angle for all subjects (44 nerves). Average values and standard errors of means are shown for each experimental point. A fitted straight line is also shown.

Figure 6 shows how the percentage of stretch as measured for the distance *d*_AE-BE_ (Figure 4), taking the straightened upper arm (0° angle of flexion) as the reference, are related to the angles of flexion at elbow. It is apparent that they almost fit a straight line (*R*^2^ = 0.9881) and it is reasonable to assume that these two parameters are proportional to each other. From this fitted straight line it was estimated that about 31% increase in stretch occurs for an angle of flexion of about 135°, corresponding to an increasing stretch rate of about 0.22% per degree of flexion.

The means of *NCV*_BE-W_, *NCV*_AE-W_ and *NCV*_AE-BE_, together with the corresponding standard errors of mean, are presented in Table 1 for different angles of flexion and corresponding percentages of stretch for both left and right hands separately. The respective *p* values obtained using Page’s L Trend test are also shown. For *NCV*_BE-W_, the *p* values (0.21 and 0.64) are much greater than the critical value (0.05) indicating a very low, almost no variation of *NCV*_BE-W_ with stretch. For *NCV*_AE-W_, the *p* values (0.01 and 0.04) are both less than the critical value, indicating a significant and systematic increase in NCV with the angle of flexion, while for *NCV*_AE-BE_, the *p* values (0.008 for both) indicate highly significant and systematic increase in NCV with the angle of flexion. Following the linear relationship between the percentage of stretch of the AE-BE segment and the angle of flexion (Figure 6), the rest of the results will be related to the to the percentage of stretch of the AE-BE segment directly, although these were obtained in terms of the angle of flexion experimentally.

**Table 1:** Average values (± SE) of segmental NCVs corresponding to different angles of flexion and different percentages of stretch.

Angle of flexion (deg)	% Stretch (Approx)	*NCV*_BE-W_ (m/s)				*NCV*_AE-W_ (m/s)				*NCV*_AE-BE_ (m/s)			
		Left hand	*p* value	Right hand	*p* value	Left hand	*p* value	Right hand	*p* value	Left hand	*p* value	Right hand	*p* value
0	0	60.5 ± 1.1	0.21	59.0 ± 1.0	0.64	57.5 ± 0.7	0.01	57.5 ± 0.7	0.04	52.7 ± 0.7	0.008	55.0 ± 1.0	0.008
45	12	62.4 ± 0.9		60.4 ± 1.0		59.6 ± 0.6		59.5 ± 0.8		55.6 ± 1.0		58.4 ± 1.2	
90	19	62.8 ± 1.1		60.5 ± 0.9		60.7 ± 0.7		60.4 ± 0.8		58.4 ± 1.1		60.9 ± 1.3	
110	25	62.3 ± 0.9		61.1 ± 1.0		61.4 ± 0.7		61.8 ± 0.8		60.5 ± 1.2		63.5 ± 1.2	
135	31	62.7 ± 1.2		60.2 ± 1.0		62.5 ± 0.8		61.9 ± 0.7		62.7 ± 1.3		65.4 ± 1.2	

Corresponding *p* values obtained using Page’s L Trend test are also shown.

**Figure 7:**
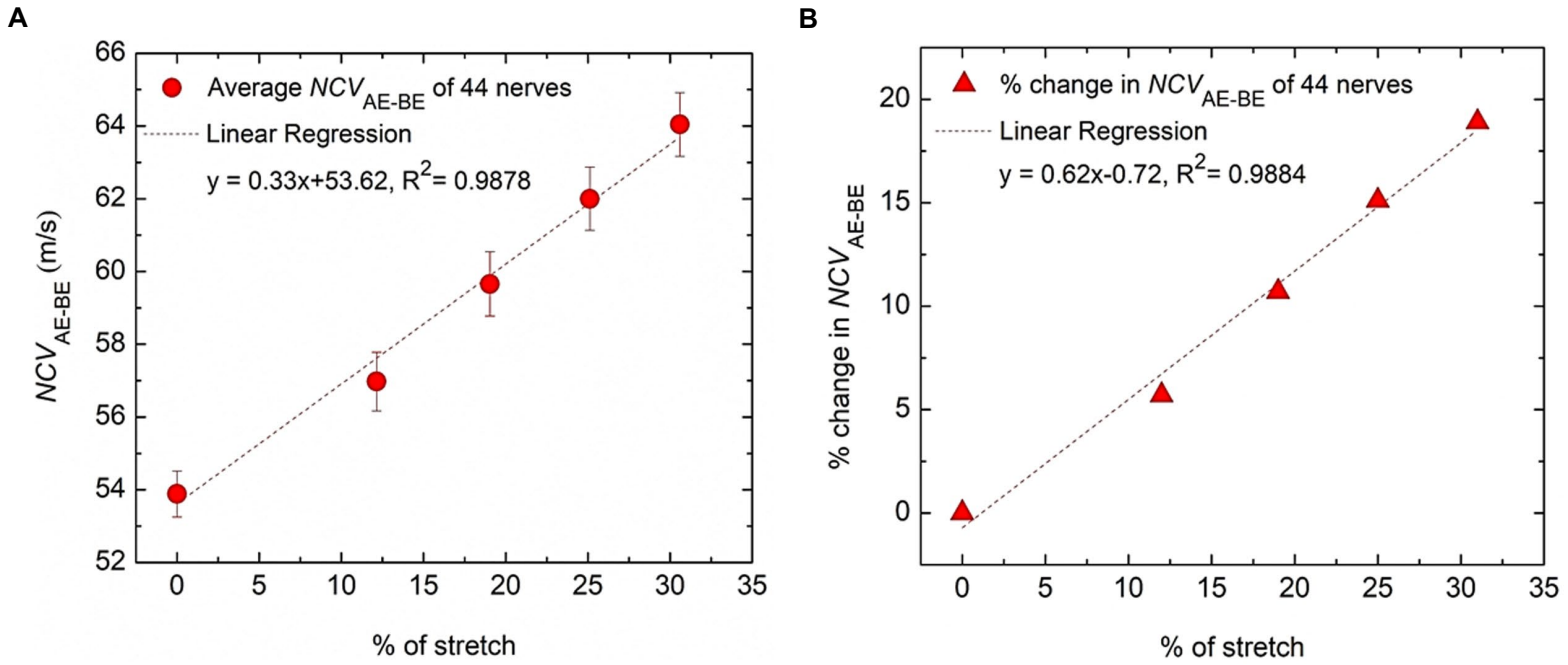
Variation of conduction velocity at the AE-BE segment. **(A)** The change of *NCV*_AE-BE_ plotted against the percentage of nerve stretch due to elbow flexion, the references of both being the corresponding values at 0° angle of flexion. **(B)** The percentage change of *NCV*_AE-BE_ plotted against the percentage of nerve stretch due to elbow flexion, the reference of the former being the corresponding value at 0% stretch. Fitted straight lines are also shown.

It can be seen from Table 1 that the differences between NCV values of the left hand and the right hand are minimal, and therefore, these were combined to obtain the average values of segmental NCVs, essentially giving a greater number of samples. Following this, the percentage increase in *NCV*_AE-BE_ corresponding to percentage of stretch are presented in Table 2. The *NCV*_AE-BE_ (this is the segment where the maximum contribution of the stretch is expected) as shown in Figure 5C against the angle of elbow flexion, are plotted in Figure 7A against the percentage of nerve stretch. Again, the percentage changes in *NCV*_AE-BE_ with reference to that at 0% stretch, as shown in Table 2, are plotted in Figure 7B against the percentage stretch of nerve. The fitted straight line graphs, their equations and the R^2^ values are also shown.

**Table 2:** Percentage increase in segmental conduction velocity, *NCV*_AE-BE_ corresponding to percentage of nerve stretch (straightened upper limb at 0° flexion taken as reference).

% nerve Stretch(Approx)	0	12	19	25	31
% increase in *NCV*_AE-BE_ (reference value at 0% stretch)	0	5.7	10.7	15.1	18.9

The trend line equation in Figure 7A has an *R*^2^ value of 0.9878 while that in Figure 7B is 0.9884, both indicating very good linear fits. The rate of change of *NCV*_AE-BE_ is about 0.33 m/s per unit percentage stretch of nerve and the rate of change in the percentage change in *NCV*_AE-BE_ is about 0.62% per unit percentage stretch of nerve. From Figure 7B it may be said that the percentage change in the NCV of a myelinated nerve is directly proportional to the percentage of stretch within the range shown (<31% stretch); corresponding to a stretch of 31% an increase of about 19% in NCV was obtained.

## Discussion

In this study, we experimentally investigated the dependence of conduction velocities of different segments of the ulnar nerve produced by elbow flexion and tried to investigate whether these support suggestions proposed earlier by Rabbani (2018) based on physics and anatomical concepts. These suggestions held that the nerve is stretched at elbow due to flexion and that the conduction velocity of myelinated nerve fibres increases with stretch. We would also discuss whether other factors are involved in the outcome of the experimental work.

Through this experiment we were able to quantify the dependence between nerve stretch (assuming no slackness to remove) and the flexion angle to a high degree of significance. This dependence is apparent from Figure 6, which shows that the percentage increase of stretch of the AE-BE segment is proportional to the angle of flexion.

The inferences related to the NCVs of the three nerve segments are supported by the *p* values obtained using Page’s L Trend and presented in Table 1. Each of the *p* values of 0.21 and 0.64 for *NCV*_BE-W_ of the two hands separately are much greater than the critical value of 0.05. Therefore, these indicate clearly that NCV of the BE-W segment remains essentially unchanged throughout the various degrees of elbow flexion (0° to 135°). However, it may be seen in Figure 5A that although *NCV*_BE-W_ remains essentially unchanged at about 61.5 m/s for higher angles of flexion, the value is slightly less (about 60 m/s) at full extension (0° flexion). A possible explanation of this slight initial increase may be a mild stretch of the proximal part of this nerve segment at small angles of elbow flexion, becoming insignificant at higher angles.

For *NCV*_AE-W_ the *p* values of 0.01 and 0.04 for the two hands are both less than the critical value of 0.05, indicating a significant increase with angle of flexion, while for *NCV*_AE-BE_ the *p* values of 0.008 for both hands indicate highly significant and systematic increase in NCV with the angle of flexion. Based on the linear relationship of nerve stretch (assuming no slackness within the measured segment) to angle of flexion (Figure 6) it may be said that NCV increases significantly with nerve stretch. The reason for the relatively higher *p* values (less systematic increase) for *NCV*_AE-W_ compared to that for *NCV*_AE-BE_ is expected since *NCV*_AE-W_ includes values for both AE-BE and BE-W segments of which the former is stretched while the latter is not.

Figure 7A shows that *NCV*_AE-BE_ is proportional to the percentage stretch of nerve, the rate being about 0.33 m/s per unit percentage nerve stretch. However, as found out by Schuind et al. (1995) and Novak et al. (2012) on cadavers, there may be a slack in the proximal segment of the nerve in the upper arm which may be removed with increasing angles of elbow flexion. This removal of slack may contribute to an apparent increase in the measured NCV at low angles of flexion through measurements outside the skin, since the actual nerve length does not change internally. Considering the measurements of Novak et al. (2012), a point on the nerve about 3 cm proximal to the pivotal point of the medial epicondyle was pulled toward the elbow by about 0.7 cm (more than 20%) on full flexion. Had there been a slack in the nerve in the medial epicondyle region, this movement would not have occurred. Again, measurements of Schuind et al. (1995) indicated a significant elongation (about 18%, between 90^o^ and 135^o^ elbow flexion) of the proximal ulnar nerve segment between about 2 cm and 6 cm from the point on the nerve at the middle of the medial epicondyle (estimated from given schematic figures and quoted numbers). Furthermore, the ultrasound scanning measurements of the cross sectional area (CSA) of the ulnar nerve in live human subjects at the medial epicondyle by Prasetyo et al. (2021) indicated reduction of CSA within a 4 cm length around the medial epicondyle due to flexion, more so at the middle than at the edges of this segment, which also supports the previous findings of Thoirs et al. (2008). Again, photographs of this nerve segment presented by Schuind et al. (1995) indicated the presence of a possible stretch in flexion. All the above suggest the possibility of the short segment around the medial epicondyle to go through stretch during elbow flexion. It may be noted that our measurement covered a distance of 6 cm in the proximal direction from the midpoint of the nerve curving around medial epicondyle and contribution of the slack should be small, particularly at large angles of flexion, leaving some room for error only at low angles of flexion. Future measurements within about 4 cm around the medial epicondyle may give a better understanding of this phenomena since this segment may have almost no slack as discussed above.

Novak et al. (2012) also reported movement of the ulnar nerve distal to elbow due to wrist extension and flexion. In the present measurement, this aspect was not looked at and should be considered in any future studies.

One may consider other factors that may have affected the results. One is the possibility of cubital tunnel compression with stretch, but this will contribute to a reduction in NCV and is not expected for healthy individuals. Stretch may restrict blood flow in nerves, but Mendonca et al. (2020) experimentally showed that up to 80% blood flow restrictions during low intensity exercise, NCV essentially remained unaffected. The physical arrangements of the intracellular cytoskeletal elements in an axon is expected to change on stretch, which may affect the conduction delay through the mechanism illustrated by Equation 1. Although it is not well known how the electrical resistance of a stretched axon will change because of the rearrangement of the intracellular cytoskeletal elements, in most possibility, these are expected to increase the axonal resistance. Even if one assumes the resistivity of the axoplasm to remain constant on stretch, reduction in the axonal diameter on stretch is expected to increase both the axonal resistance and the capacitances involved, which are expected to increase the conduction delay as indicated by Equation 1, resulting in a reduction in conduction velocity. However, as explained by Rabbani (2018), the axonal resistance as given in Equation 1 will be of importance for conduction in unmyelinated fibres only; it will have negligible contribution in a myelinated fibre, where the newly introduced resistance *R*_ne_ will dominate.

Again, ion channels, particularly of the recently discovered mechanosensitive two-pore-domain potassium channels (K2P: TRAAK and TREK-1) that are found at the nodes in afferent fibres, are expected to increase conduction delay, eventually leading to conduction block (Kanda et al., 2019; Liu et al., 2021; Schwarz, 2021). A recent experimental measurement by Kanda et al. (2023) clearly showed that mechanical stretch decreases the resting potential (makes it more negative). This increases the potential gap to reach the threshold potential needed for depolarization, thus decreasing the excitability of the nerve fibre and increasing the conduction delay (it takes longer for the transmembrane capacitance to charge through the greater potential range). Therefore, all of the above agents tend to reduce the CV of nerve fibres and none of these may explain the increase in conduction velocity with stretch as experimentally observed. Histopathological images of human peroneal and tibial nerves show that there are wavy patterns of nerve fibres within a nerve trunk (Kerns et al., 2019) which straighten up when stretched to conduction failure. Therefore, removal of slacks in the nerve trunk and unfolding of the wavy patterns of the nerve fibres within a nerve trunk both may contribute partly to the apparent increase in NCV with stretch, which actually do not need any change in any conduction parameters. However, the above is expected to hold at low angles of elbow flexion but not for greater angles. Besides, in the experiments by Kanda et al. (2019, 2023) where they stretched a nerve *in vitro*, possibility of a gross nerve slack may be ruled out, leaving only the unfolding of the wavy patterns. Again, comparing the morphology of unmyelinated and myelinated axons, it may be envisaged that an unmyelinated axon is more liable to have such wavy patterns than the myelinated axons. Combining all the factors discussed above, the new mechanism proposed by Rabbani (2018) appears to be an important and plausible explanation of the observed increases in ulnar NCV under stretch.

At this point the work presented by Liu et al. (2021) is worth going into details as it presents a very carefully designed experimental work on individual afferent nerve axons of both myelinated A-fibres and unmyelinated C-fibres. This work was carried out *in-vitro* on dissected rat sciatic nerves, measuring conduction delays in single axons (as against whole nerve trunks by many other authors, which carry some uncertainties) due to different magnitudes of stretch. They found that for both A-fibres (10 samples) and C-fibres (12 samples) the conduction delay increased immediately and also reverted back to the initial value immediately if the stretch was limited to a certain value, which was about 30% in myelinated A-fibres and 20% in unmyelinated C fibres (the values estimated from the graphs shown). Within these respective limits, the conduction delay increased linearly with increasing stretch, but interestingly, the percentage increase of delay in relation to the percentage increase in nerve length in A-fibres was almost half that in C-fibres, comparing the mean values. However, the authors mistakenly interpreted this as decrease of conduction velocity (CV) for both. This interpretation is correct for the C-fibres but not for the A-fibres and the following explanation will clarify the point.

The authors of the paper drew graphs of the percentage increase in conduction delay against ‘stretch ratio’, the latter being defined as a ratio of the length of a stretched nerve to that of the unstretched nerve (for example, a stretch ratio of 1.1 corresponding to a 10% increase of length). The numerical values of the slopes of the fitted straight lines were 85 and 158 for the A-fibres and the C-fibres, respectively. If the ‘stretch ratio’ was converted to percentage increase in nerve length from an unstretched condition, this would mean that for a 1% increase in nerve length the conduction delay increased by 0.85% for A-fibres, and by 1.58% for C-fibres. Since CV is given by the ratio of nerve length to conduction delay, there would be no change in CV if the conduction delay increases by 1% corresponding to a 1% increase in nerve length, which now becomes the reference. Therefore, an increase of conduction delay by 0.85% will mean that the delay was less than that for the case where there is no change in CV. This implies that the CV, in effect, has increased by 0.15% (=1–0.85). On the other hand, for the C-fibres, an increase of conduction delay by 1.58% will mean that the delay was more than that for the case where there is no change in CV. This implies that the CV, in effect, has decreased by 0.58% (=1.58–1).

Now one may invoke all the factors that increase or decrease the measured CV as mentioned above to explain the 0.58% decrease in CV in unmyelinated C-fibres that obviously does not relate to the new mechanism proposed by Rabbani (2018), which holds for myelinated fibres only. This brings an important question as to the 0.15% increase in CV for myelinated A-fibres which occurs over and above the 0.58% reduction in CV for unmyelinated fibres. Assuming all the factors discussed above to remain the same for both unmyelinated and myelinated fibres (ignoring any differences due to different fibre diameters of those measured), one has to explain the greater range of increase of 0.73% (=0.58% + 0.15%) for myelinated fibres on stretch. Considering the status of the current knowledge, the only candidate for this increase in CV is the new mechanism proposed by Rabbani (2018) and briefly presented in the introduction. This mechanism is capable of explaining such a large increase in CV based on the sharp reduction in *R_ne_* that occurs because of increasing the gap between the interdigitated protrusions of the myelin sheath at the node due to stretch.

In the results section it was mentioned that the percentage change in *NCV*_AE-BE_ that we found through our measurements was about 0.62% per unit percentage stretch of nerve which is more than 0.15% obtained by Liu et al. (2021), as obtained through our reanalysis above. This may be due to the fact that our experimental values were obtained for efferent Aα-fibres while those by Liu et al., was obtained for afferent Aβ-fibres. As mentioned above, although the abundant presence of TRAAK and TREK-1 channels were found in the nodes of afferent Aβ-fibres their presence has not been confirmed in efferent Aα-fibres. Since on stretch TRAAK and TREK-1 channels may contribute to a reduction in CV, this factor was essentially absent in efferent Aα-fibres, for which a higher value of 0.62% was obtained. Of course, we need to consider the limitations of our experiment which has inherent uncertainties in pin pointing nerve stimulation points from skin positions, and the contribution of slack removal and unfolding of wavy patterns at low values of stretch.

Now, focusing on the starting and ending values of *NCV*_AE-W_ and *NCV*_AE-BE_, which stand at 57.5/62.5 and 54/64, respectively, in units of m/s, and comparing these with the steady value of 61.5 m/s for *NCV*_BE-W_, with slightly lower value, 59.7 m/s at 0^o^ flexion, we see that the starting values of both *NCV*_AE-W_ and *NCV*_AE-BE_ are lower than the steady *NCV*_BE-W_ value while the ending values are higher. Previous works (Harding and Halar, 1983; Sattari and Emad, 2007) also had similar outcomes. We think this is a very interesting observation and needs to be explained, for which we would like to invoke again the new mechanism proposed by Rabbani (2018). Here, for simplicity, we assume that NCV is the same throughout the upper limb for the ulnar nerve, except at the elbow region where flexion and consequent stretching takes place. At 0° flexion, the *NCV*_AE-BE_ is 54 m/s, which may be taken to correspond to the value for an unstretched nerve. Since according to Rabbani (2018) NCV increases with stretch, the higher value of *NCV*_BE-W_ of 59.7 m/s at 0° flexion (increasing to about 61.5 m/s at higher angles of flexion) indicate the possibility of this nerve segment to be under a state of stretch always. Although one may suggest whether this apparent increase in NCV is due to errors in nerve length measurements as these were taken from outside the skin, the errors should be small for the long BE-W segment. Besides, as discussed above, of the 10 cm AE-BE segment around the medial epicondyle, a segment of 6 cm was on the proximal side for which the effect of slack would be small, particularly at greater angles of flexion. However, this particular observation needs to be looked into further.

## Conclusion

1. Through measurements outside the skin, we have experimentally shown that the magnitude of ulnar nerve stretch at elbow is directly proportional to the angle of elbow flexion. Through logical arguments we claim that except for some errors at low values of flexion, this relationship generally holds.
2. We have experimentally shown that the NCV of ulnar nerve obtained using evoked EMG from ADM muscle in the palm, which involves myelinated efferent nerve fibres only, increases with nerve stretch that cannot be explained based on other known factors except the new mechanism proposed recently by Rabbani (2018) and presented in the introduction.
3. We have extended the work further quantifying the above relationship through careful design of an experiment and have shown that the percentage increase in NCV of a myelinated nerve is directly proportional to the percentage of stretch, at least up to a certain limit. Our experiments show an increase of about 19% in the motor NCV of the ulnar nerve at the elbow for about 31% stretch. However, there may be some error at low angles of flexion due to slackness of the nerve trunk and wavy patterns of fibres within a nerve trunk.
4. We have re-analyzed experimental data presented by Liu et al. (2021) to show that the an immediate and significant increase in the CV of myelinated fibres may only be explained on the basis of the new mechanism put forward by Rabbani (2018). It is to be noted that this explanation achieved a greater strength since the above experiments compared the measured values on both myelinated and unmyelinated nerve fibres (single axons) under exactly the same measurement conditions.
5. From our experimental results, we may infer that, the ulnar nerve in the lower arm (BE-W segment) is normally maintained under slight stretch, which help speed up nerve propagation.

Further measurements on ulnar nerve, focusing within about a 4 cm segment around the medial epicondyle needs to be performed to give a better understanding and quantification of the suggested NCV increase with nerve stretch. Again, Distribution of F-Latency (DFL) measurements (Rabbani et al., 2007; Rabbani, 2011) on the ulnar nerve for different angles of elbow flexion may help isolate some delay mechanisms since in this case one is not working on a nerve segment only, rather the measurement will involve the whole nerve length, right from the axon hillock in the spinal cord to the connected muscle in the palm. If there are issues due to removal of slackness of nerve trunks or unfolding of wavy patterns of fibres within a nerve trunk, these are not supposed to affect the conduction delay in this measurement.

Further work needs to be done in quantifying the new proposed resistance *R*_ne_ (Rabbani, 2018) between the outside of the nodal membrane and extracellular fluid, which appears to be a major mechanism controlling nerve conduction in myelinated nerves and was successful in explaining some anomalous experimental observations; predictions based on which were supported by the present study.

## Data availability statement

The raw data supporting the conclusions of this article will be made available by the authors, without undue reservation.

## Ethics statement

The studies involving human participants were reviewed and approved by Bangladesh Medical Research Council (No. 36817122020). The patients/participants provided their written informed consent to participate in this study.

## Author contributions

SS and KR contributed to the conception of the project idea and project planning. SS designed the experimental setup, performed experiments, data analysis, and statistics, and composed the first draft of the manuscript. MK, ZM, and KR provided significant inputs to the data analysis. SS, MK, ZM, and KR interpreted the results. All authors, especially KR contributed to and critically revised the final manuscript and have approved the content for publication and agreed to be accountable for all aspects of the work in ensuring that questions related to the accuracy or integrity of any part of the work are appropriately investigated and resolved.

## Funding

This work was supported partly by the grants from the Directorate of Advisory, Extension and Research Services (CASR), BUET, Bangladesh [No. 348(3)], and Basic Research Grant of BUET (No. Est/R-60/Re-5336).

## Conflict of interest

The authors declare that the research was conducted in the absence of any commercial or financial relationships that could be construed as a potential conflict of interest.

## Publisher’s note

All claims expressed in this article are solely those of the authors and do not necessarily represent those of their affiliated organizations, or those of the publisher, the editors and the reviewers. Any product that may be evaluated in this article, or claim that may be made by its manufacturer, is not guaranteed or endorsed by the publisher.
